# Supplementary material for: Organic and Conventional Coffee Beans, Infusions, and Grounds as a Rich Sources of Phenolic Compounds in Coffees from Different Origins
Source: Molecules. 2025 Mar 13;30(6):1290. doi: 10.3390/molecules30061290 (PMC11946014; doi:10.3390/molecules30061290)
Supplement: Supplementary file 1 [file molecules-30-01290-s001.zip › molecules-3518033-supplementary.pdf]

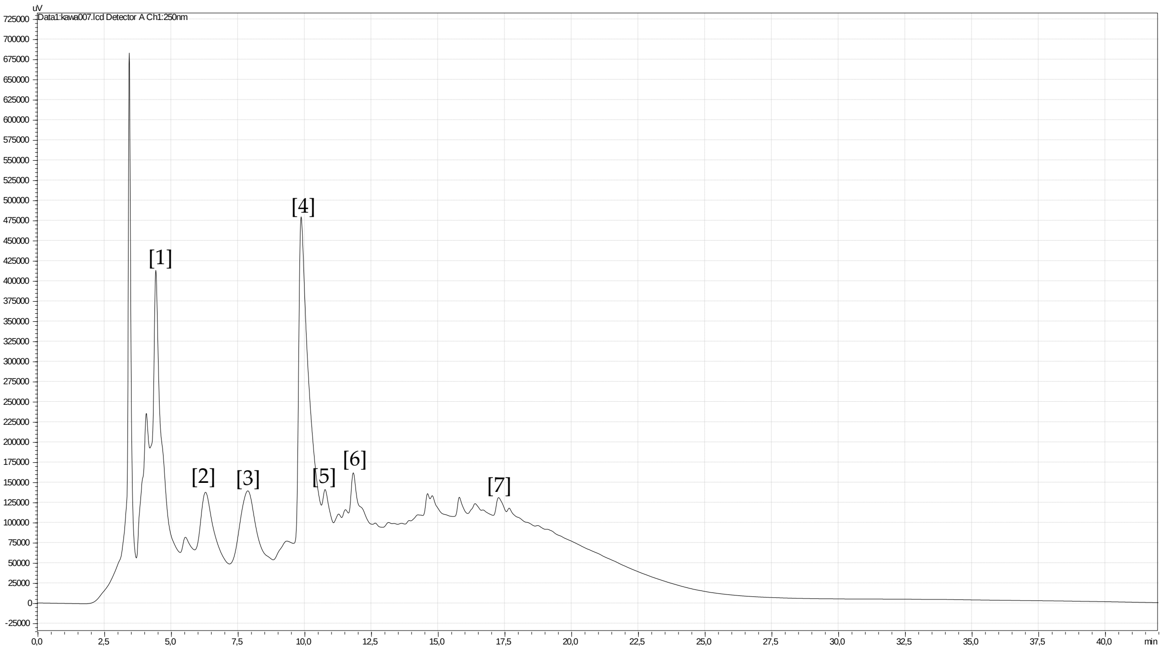

Figure S1. The example of chromatogram of identified phenolic compounds and caffeine in organic coffee beans from Peru: (1) gallic acid, (2) epigallocatechin, (3) catechin, (4) caffeine, (5) chlorogenic acid, (6) caffeic acid, (7) quercetin

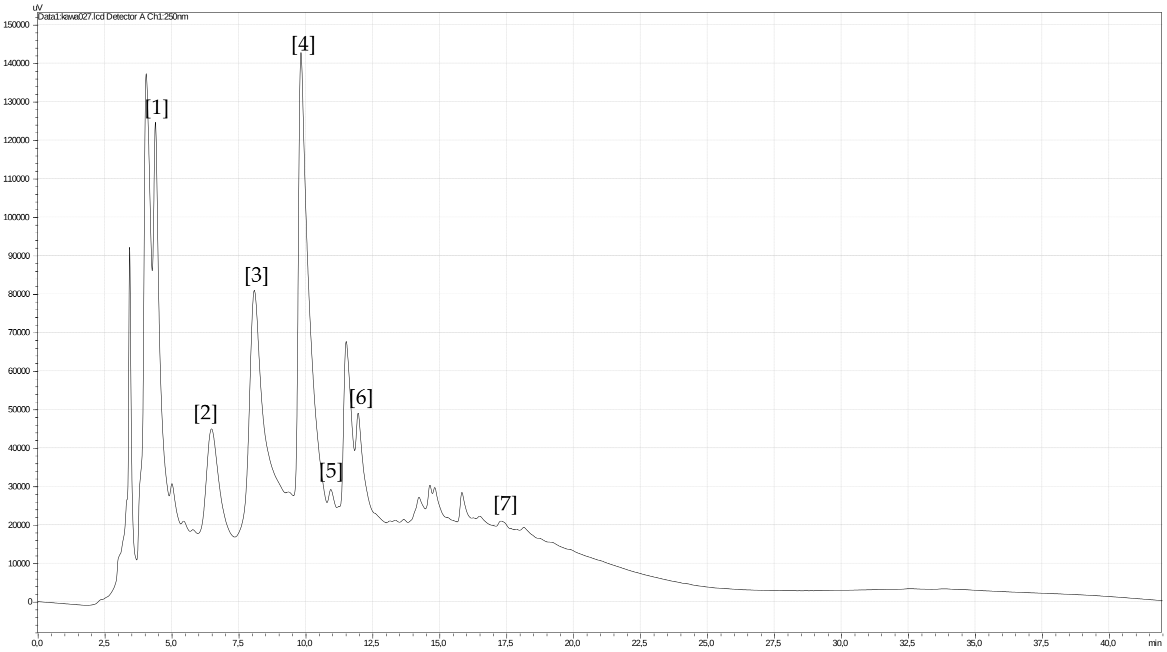

Figure S2. The example of chromatogram of identified phenolic compounds and caffeine in organic coffee brew from Peru: (1) gallic acid, (2) epigallocatechin, (3) catechin, (4) caffeine, (5) chlorogenic acid, (6) caffeic acid, (7) quercetin

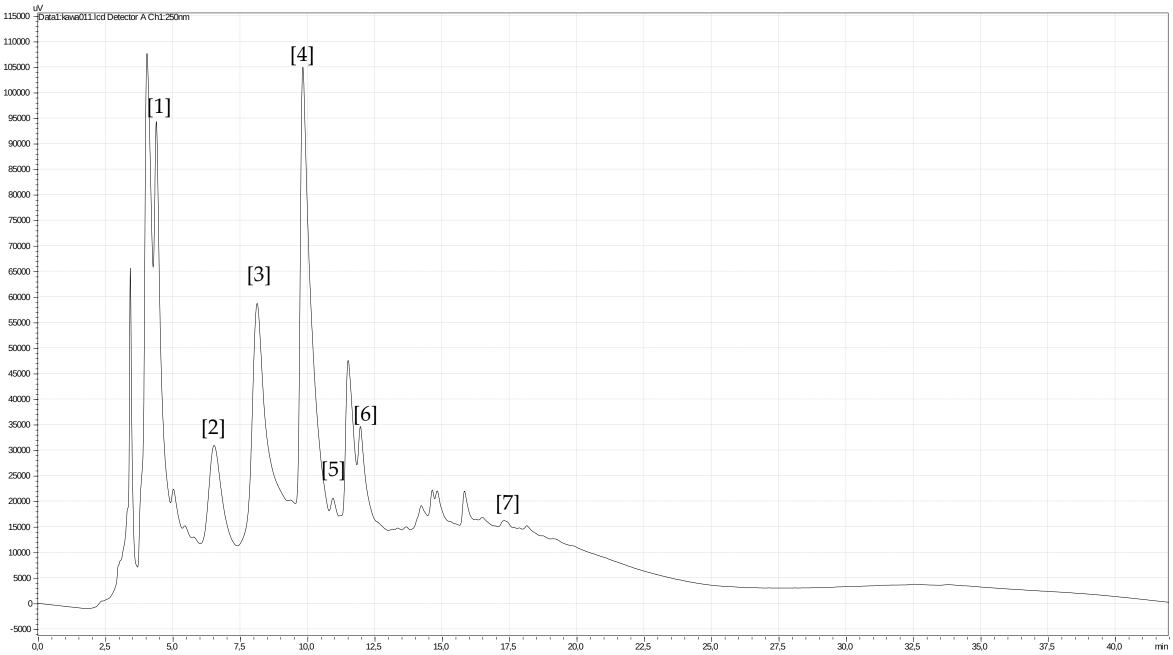

Figure S3. The example of chromatogram of identified phenolic compounds and caffeine in organic coffee grounds from Peru: (1) gallic acid, (2) epigallocatechin, (3) catechin, (4) caffeine, (5) chlorogenic acid, (6) caffeic acid, (7) quercetin

Table S1. The natural condition in coffee plantation (according to coffee producers information)

	**temperature**	**air humidity**	**crop heigh**	**rainfall**
Peru	15–24°C	56%-76%	1200- 2000 m above sea level	0.3-100 mm
Sumatra	15–25°C	66%-78**%**	700- 1500 m above sea level	5-1000 mm
Ethiopia	20–25°C	54%-67%	1000- 2100 m above sea level	0-100 mm
